# Modification of CCNU pharmacokinetics by misonidazole--a major mechanism of chemosensitization in mice.

**DOI:** 10.1038/bjc.1983.104

**Published:** 1983-05

**Authors:** F. Y. Lee, P. Workman

## Abstract

We have investigated the effect of misonidazole (MISO) on the pharmacokinetics of 1-(2-chloroethyl)-3-cyclohexyl-1-nitrosourea (CCNU) in mice. CCNU and its monohydroxylated metabolites were measured using a high performance liquid chromatography (HPLC) method. In the absence of MISO the plasma disappearance of CCNU was biphasic with a t 1/2 alpha of 2.3 min and a t 1/2 beta of 53 min. The monohydroxylated metabolites of CCNU also followed biphasic clearance kinetics. A large single dose of MISO (0.5 mg g-1), given i.p. 30 min prior to CCNU, prolonged the t 1/2 alpha by a factor of 2.6 but had no effect on t 1/2 beta. In addition, the apparent volume of distribution was decreased by a factor of 1.6. Consequently, the plasma area under the curve (AUC0 - infinity) was increased by a factor of 1.7 for CCNU and by a factor of 2.0 for total nitrosourea (CCNU + monohydroxylated metabolites). The effects of MISO on CCNU kinetics were dependent on MISO dose and plasma concentration and on the interval between MISO and CCNU administration. The concentration of CCNU was measured in 4 tumours: the KHT, RIF-1 and EMT6 mouse tumours, and the HT29 xenograft. For all 4 tumours, 0.5 mg g-1 MISO raised the tumour concentrations of CCNU and total nitrosourea by a considerable amount (2-2.5 times). More detailed studies in the KHT tumour demonstrated that there was a significant lag period before peak tumour CCNU concentrations were reached, and that MISO increased the peak concentrations by a factor of about 2.4. In contrast, there was no such lag period for the plasma and MISO did not increase the plasma peak CCNU concentrations. These data strongly suggest that modification of the pharmacokinetics may be a major contributory factor in the enhancement of CCNU cytotoxicity by large single doses of MISO in vivo.


Br. J. Cancer (1983), **47**, 659–669

### Modification of CCNU pharmacokinetics by misonidazole— A major mechanism of chemosensitization in mice

F.Y.F. Lee & P. Workman

*MRC Clinical Oncology and Radiotherapeutics Unit, MRC Centre, Hills Road, Cambridge CB2 2QH.*

**Summary** We have investigated the effect of misonidazole (MISO) on the pharmacokinetics of 1-(2-chloroethyl)-3-cyclohexyl-1-nitrosourea (CCNU) in mice. CCNU and its monohydroxylated metabolites were measured using a high performance liquid chromatography (HPLC) method. In the absence of MISO the plasma disappearance of CCNU was biphasic with a $t_{\frac{1}{2}\alpha}$ of 2.3 min and a $t_{\frac{1}{2}\beta}$ of 53 min. The monohydroxylated metabolites of CCNU also followed biphasic clearance kinetics. A large single dose of MISO ($0.5\,mg\,g^{-1}$), given i.p. 30 min prior to CCNU, prolonged the $t_{\frac{1}{2}\alpha}$ by a factor of 2.6 but had no effect on $t_{\frac{1}{2}\beta}$. In addition, the apparent volume of distribution was decreased by a factor of 1.6. Consequently, the plasma area under the curve ($AUC_{0-\infty}$) was increased by a factor of 1.7 for CCNU and by a factor of 2.0 for total nitrosourea (CCNU+monohydroxylated metabolites). The effects of MISO on CCNU kinetics were dependent on MISO dose and plasma concentration and on the interval between MISO and CCNU administration. The concentration of CCNU was measured in 4 tumours: the KHT, RIF-1 and EMT6 mouse tumours, and the HT29 xenograft. For all 4 tumours, $0.5\,mg\,g^{-1}$ MISO raised the tumour concentrations of CCNU and total nitrosourea by a considerable amount (2–2.5 times). More detailed studies in the KHT tumour demonstrated that there was a significant lag period before peak tumour CCNU concentrations were reached, and that MISO increased the peak concentrations by a factor of about 2.4. In contrast, there was no such lag period for the plasma and MISO did not increase the plasma peak CCNU concentrations. These data strongly suggest that modification of the pharmacokinetics may be a major contributory factor in the enhancement of CCNU cytotoxicity by large single doses of MISO *in vivo*.

Many studies have shown that electron affinic radiation sensitizers such as misonidazole (MISO) can enhance the cytotoxic efficacy of a number of anti-cancer agents against tumours in mice (for review see McNally, 1982; Siemann, 1982*a*). In most cases, the enhancements were greater in tumours than in normal tissues resulting in a therapeutic gain. A number of clinical trials are now in progress.

The mechanism of action by which MISO exerts its chemosensitising effect is unknown, although a number of possible mechanisms have been discussed (Brown, 1982; Millar, 1982; Siemann, 1982*a*). In view of the selective enhancement of tumour toxicity, most investigators have favoured hypoxia-mediated mechanisms acting at the cellular level, e.g. inhibition of DNA repair processes or depletion of intracellular thiols. However, studies in our laboratory have shown that MISO also inhibits the hepatic drug-metabolising enzymes suggesting that the mechanism may involve changes in cytotoxic drug pharmacokinetics (Workman & Twentyman 1982, Workman *et al.*, 1983). Some evidence in favour of altered pharmacokinetics has been obtained for melphalan (Clutterbuck *et al.*, 1982), BCNU and cyclophosphamide (Tannock, 1980), and chlorambucil (Workman *et al.*, 1983), but a pharmacokinetic mechanism for therapeutic gain has not yet been provided. We now present data which show that MISO profoundly alters the metabolism of CCNU and its active metabolites in a way which appears to explain the chemosensitization and therapeutic gain obtained with large doses of MISO in mice.

Correspondence: F.Y.F. Lee

Received 2 November 1982; accepted 2 February 1983.

### Materials and methods

### *Drugs*

MISO was supplied by Roche Products Ltd. and CCNU by the Drug Synthesis and Chemistry Branch of the National Cancer Institute, USA, and by Lundbeck. The synthetic monohydroxylated-CCNU metabolites were kindly given by Dr. T.P. Johnston of the Southern Research Institute, Alabama, U.S.A.

### *Mice and tumours*

Inbred female C3H/He and male BALB/c mice were supplied by OLAC. Male CBA nude mice were supplied by NIMR (Mill Hill). KHT and RIF-1 tumours were grown in C3H mice, EMT6 tumours in BALB/c mice and HT29 xenografts in CBA nude

© The Macmillan Press Ltd., 1983

mice. KHT, RIF-1 and EMT6 tumours were grown in the gastrocnemius muscles of the hind leg as described by Twentyman *et al.* (1979). The HT29 human colonic adenocarcinoma xenograft was grown bilaterally from s.c. injections in the flank as described for the HT29R line by Warenius & Bleehen (1982). Unless otherwise stated data reported are for C3H/He mice.

*Drug administration*

CCNU was first dissolved in a 1:1 mixture of ethanol/Cremophor-EL (Sigma) and then diluted 1:4 with saline. In this form CCNU remains stable for at least 8 h at room temperature as determined by high-performance liquid chromatography (HPLC) assay. This solution was injected in $0.01\,\mathrm{ml\,g^{-1}}$ body weight.

MISO was dissolved in Hanks' balanced salt solution and injected i.p. in $0.04\,\mathrm{ml\,g^{-1}}$ body weight.

In most experiments mice received $0.5\,\mathrm{mg\,g^{-1}}$ ($2.5\,\mathrm{mmol\,kg^{-1}}$) MISO followed by an appropriate dose of CCNU 30 min later. In some experiments a range of MISO doses ($0.1$–$1.0\,\mathrm{mg\,g^{-1}}$) was used, while in others CCNU was given at varying intervals (0.5–6 h) after MISO. In all instances controls were given the appropriate vehicles.

*Sample preparation*

Blood was drawn by heart puncture into heparinized syringes (0.5–0.8 ml per mouse). This was immediately cooled on ice and pooled with blood from mice of the same group. The pooled blood was immediately centrifuged in a refrigerated Du Pont Sorvall RC-5B Superspeed Centrifuge (Du Pont Instruments, U.S.A.) at 4000 g for 10 min. For CCNU analysis, aliquots of plasma were extracted with an equal volume of cold diethyl ether (HPLC grade, Fisons). Aliquots of the supernatant were evaporated to dryness *in vacuo* using a Savant Speed Vac Concentrator coupled to a Model 100A Refrigerated Condensation Trap (Uniscience). The dry residues were redissolved in $50\,\mu l$ ethanol (Spectrograde, Fisons) and stored sealed at $-20°C$. $35\,\mu l$ of the ethanol concentrate was used for HPLC analysis.

Tumours were excised rapidly and immediately frozen to $-70°C$ in an ethanol/dry ice freezing mixture. A 20% (w/v) homogenate in distilled water was then prepared using a 'Verso' Laboratory Mixer Emulsifier (Silverson, U.K.). The homogenate was then processed as for plasma.

Recovery from plasma and tumour homogenate was consistently 100% for CCNU and 85% for the major, *trans*-4-monohydroxylated metabolite, as measured by HPLC. The recoveries were not affected by MISO.

For MISO analysis aliquots of plasma were precipitated with 4 vols of methanol (HPLC grade, Rathburns) and cooled on dry ice. Following centrifugation at 4000 g for 10 min in a Sorvall RC-5B Centrifuge, aliquots of supernatant were removed for HPLC analysis.

*High-performance liquid chromatography*

Reversed-phase HPLC was used for both MISO and CCNU analyses. The HPLC equipment (Waters Associates, Milford, Mass., U.S.A.) consisted of Model 6000A chromatography pumps, Model 710B Automated Sample Processors (WISP), Data Module, Model 720 System Controller, RCM-100 Radial Compression Modules, and Model 440 u.v. absorbance detectors. Separations were carried out on Waters Radial-PAK reverse-phase bonded octadecylsilane (C18) cartridge columns (8 mm I.D., $5\,\mu m$ or $10\,\mu m$ diameter spherical particles) protected by Waters RCSS Guard—PAK C18 guard columns.

Analysis of MISO and its O-demethylated metabolite Ro 05-9963 was essentially as described previously (Workman *et al.*, 1978) but with minor modifications for use with the Radial Compression Module.

For CCNU analysis, samples were eluted by running a two-step linear gradient commencing, at the time of injection, with an initial condition of 34% acetonitrile (HPLC low u.v. absorbance grade, Rathburns) in water (HPLC grade, Fisons) and proceeding to 44% over 5 min and then to 64% over another 7 min. Absorbance was monitored at 254 nm.

With the above method the coefficients of variation were 5.9% and 7.7% for *trans*-4-hydroxy CCNU and CCNU respectively at a concentration of $0.5\,\mu\mathrm{g\,ml^{-1}}$. The lower limit of detection was approximately $10\,\mathrm{ng\,ml^{-1}}$.

Drugs and metabolites were identified by co-chromatography with authentic synthetic standards where available.

*Protein binding*

For *in vivo* binding studies, mice, with or without MISO ($0.5\,\mathrm{mg\,g^{-1}}$) pretreatment, were sacrificed 15 min after i.p. injection of CCNU ($20\,\mathrm{mg\,kg^{-1}}$) and the plasma from 4 mice was pooled. Separation of plasma water from plasma protein was accomplished by ultrafiltration across YMB membranes using the Amicon Micropartition System (Amicon, Woking, U.K.) in an MSE Chilspin Centrifuge operating at 2000 g. The ultrafiltrate obtained was analysed as described above for plasma. For *in vitro* binding studies plasma containing $2.5\,\mu\mathrm{g\,ml^{-1}}$ CCNU was incubated at 37°C for 15 min. Binding was then determined as above.

*Spontaneous chemical degradation*

CCNU was incubated at 37°C at a concentration of 5 $\mu g\, ml^{-1}$ in 0.1 M sodium phosphate buffer, pH 7.4, with or without MISO (0.5 $mg\, ml^{-1}$). Concentrations of CCNU remaining at various times were determined by HPLC.

*Pharmacokinetic parameters*

When appropriate, i.e. in regions where exponential decays operated, best fit lines were estimated by least squares regression analysis yielding half-lives with 95% confidence limits. CCNU data were fitted to the two compartment open model using the method of curve stripping (Workman & Brown, 1981). Pharmacokinetic parameters were calculated as described previously (Workman & Brown, 1981; White & Workman, 1980). Values estimated for $Vd_{area}$ and clearance are apparent values assuming 100% bioavailability. Degree of significance was calculated by Student's t-distribution.

### Results

*Pharmacokinetics of MISO in mice*

Figure 1 shows the typical pharmacokinetics of MISO given i.p. at a dose of 0.5 $mg\, g^{-1}$. CCNU, at a dose of 20 $mg\, ml^{-1}$ i.p. administered 0.5 h following MISO, had no significant effect on MISO pharmacokinetics. The plasma decay curve gives an apparent $t_{\frac{1}{2}}$ value (Workman, 1980*a*) of 0.95 (0.86–1.02) h for mice also given CCNU, compared to 0.99 (0.87–1.06) h for the controls ($P>0.1$). Concentrations of the metabolite Ro 05-9963 were also unaffected (Figure 1).

*HPLC of CCNU and its metabolites*

CCNU is metabolized rapidly by the liver microsomal mixed function oxidase system to ring-monohydroxylated products (Hilton & Walker 1975). Five of the 6 possible isomeric metabolites were detected in the plasma of our mice. Figure 2 is a representative HPLC chromatogram of the ether extract of plasma obtained from mice treated with 20 $mg\, kg^{-1}$ CCNU i.p. Peak 6 in Figure 2 is CCNU. Peaks 1 and 5 co-chromatograph with authentic standards of the *trans*-4 and *trans*-2 hydroxy CCNU isomers respectively. Montgomery *et al.* (1976) using a very similar HPLC system reported that the elution of the *cis-trans* pairs of monohydroxylated CCNUs occurs in the order of alternating diequatorial and axial-equatorial

**Figure 1** The effect of 20 $mg\, kg^{-1}$ CCNU on the pharmacokinetics of 0.5 $mg\, g^{-1}$ MISO and its metabolite Ro 05-9963 ●, ▲ MISO and Ro 05-9963 alone respectively. ○, △ MISO and Ro 05-9963 respectively with CCNU pretreatment. Each datum point is for 3 mice.

isomers which reflects their partition coefficients: thus the order is *trans*-4-hydroxy, *cis*-4-hydroxy, *cis*-3-hydroxy, *trans*-3-hydroxy, *trans*-2-hydroxy and *cis*-2-hydroxy CCNU. We have therefore assigned peaks 2, 3 and 4 as the *cis*-4, *cis*-3 and *trans*-3-hydroxy CCNU respectively.

*Pharmacokinetics of CCNU and the effects of MISO pretreatment*

Figure 3 shows all the data obtained on the plasma pharmacokinetics of CCNU in mice. Absorption of an i.p. administered dose (20 $mg\, kg^{-1}$) was extremely rapid, with peak plasma concentration being reached within 2 min after injection. The plasma clearance of CCNU follows biexponential kinetics, with a rapid initial ($\alpha$) phase followed by a slower terminal ($\beta$) phase. The $\alpha$ and $\beta$ components are normally regarded as the distribution and elimination phases respectively. In this case a considerable amount of metabolism occurs during the $\alpha$-phase (see later), and the terminal elimination may be limited by the rate of redistribution of CCNU from tissue depots back into the plasma.

Figure 3 also shows the effects of 0.5 $mg\, g^{-1}$ MISO, given 30 min before, on the plasma

**Figure 2** HPLC chromatogram of CCNU and the metabolites in an ether extract of plasma obtained 15 min after CCNU administration. Peak 1: *trans*-4-hydroxy-CCNU, peak 2: *cis*-4-hydroxy-CCNU, peak 3: *cis*-3-hydroxy-CCNU, peak 4: *trans*-3-hydroxy-CCNU, peak 5: *trans*-2-hydroxy-CCNU, peak 6: CCNU. The upper section shows the gradient profile.

pharmacokinetics of CCNU. The pharmacokinetic parameters for CCNU are summarised in Table I. The effect was highly reproducible with similar results obtained in all 4 repeat experiments. The half-life of the α phase ($t_{\frac{1}{2}\alpha}$) was increased from 2.3 (1.9–2.8) min to 5.8 (5.0–6.9) min ($P<0.001$), whereas the half-life of the β phase ($t_{\frac{1}{2}\beta}$) was not significantly changed ($P>0.1$), i.e. 53 (40.0–76.0) min as compared with 56.1 (43.1–80.2) min in the controls. However, the apparent volume of distribution (App. $Vd_{area}$), a theoretical volume in which a drug would be distributed in the body at the same concentration as in the plasma, was decreased by a factor of 1.6. The plasma area under the curve ($AUC_{0-\infty}$) for CCNU was increased by a factor of 1.7.

### *Effects of MISO pretreatment on the disposition of CCNU metabolites*

Five monohydroxylated metabolites were detected, of which the *trans*-4-hydroxy and *cis*-4-hydroxy metabolites represent ~90% of the total. The *trans*-2-hydroxy metabolite was present in trace amounts (0.03 $\mu g\,ml^{-1}$) and only in early time points and will not be considered further. The *cis*-2-hydroxy metabolite was not detected, and if present was below a concentration of 0.01 $\mu g\,ml^{-1}$.

Metabolites were detected as early as 1 min after CCNU administration. All 4 important metabolites have a similar pattern of disposition (Figure 4). Maximum concentrations were reached within 10–20 min following i.p. injection of 20 $mg\,kg^{-1}$ CCNU. Thus most of the metabolism occurs during the α-phase of CCNU clearance. The plasma

**Table I** Effect of 2.5 $mmol\,kg^{-1}$ MISO on the pharmacokinetic parameters of CCNU (20 $mg\,kg^{-1}$). MISO was given 30 min before CCNU. Calculated from pooled data from 4 experiments

| *Parameters* | *Control**† | | *MISO**† | |
|---|---|---|---|---|
| A ($\mu g ml^{-1}$) | 7.55 | (4.70–11.89) | 3.79 | (2.77–5.19) |
| B ($\mu g ml^{-1}$) | 0.190 | (0.110–0.331) | 0.406 | (0.256–0.645) |
| α ($min^{-1}$) | 0.307 | (0.249–0.365) | 0.119 | (0.100–0.139) |
| β ($min^{-1}$) | 0.013 | (0.011–0.017) | 0.012 | (0.009–0.021) |
| $t_{\frac{1}{2}\alpha}$ (min) | 2.3 | (1.9–2.8) | 5.8 | (5.0–6.9) |
| $t_{\frac{1}{2}\beta}$ (min) | 53.0 | (40.0–76.0) | 56.1 | (43.1–80.2) |
| $AUC_{0-\infty}$ ($\mu g ml^{-1} min$) | 43.0 | | 74.0 | |
| App. Clearance ($l\,kg^{-1} min^{-1}$) | 0.463 | | 0.269 | |
| App. $Vd_{area}$ ($l\,kg^{-1}$) | 35.6 | | 22.4 | |

*The values were fitted to the two compartment open model: $C_t = Ae^{-\alpha t} + Be^{-\beta t}$.
†95% confidence limits in parentheses.

**Figure 3** The effect of 0.5 mg g$^{-1}$ MISO given 30 min before on the pharmacokinetics of CCNU. ◆, ●, ▲, ▼, ■ 20 mg kg$^{-1}$ CCNU alone. ◇, ○, △, ▽, □ 20 mg kg$^{-1}$ after MISO. The data were obtained in 5 independent experiments, indicated by different symbols. Each datum point is for 3 mice.

clearance of the metabolites was biphasic in the control mice. Values for the $t_{\frac{1}{2}\alpha}$ of the metabolites could not be calculated with any precision because of the scatter in the data due to their rapid appearance and initial clearance. Values for $t_{\frac{1}{2}\beta}$ and $AUC_{0-\infty}$ are summarised in Table II.

Following MISO administration the clearance of the main *trans*-4 hydroxy metabolites and of the total nitrosoureas appeared to become monophasic, whereas the clearance of the other . metabolites remained biphasic (Figure 4). Concentrations of all the metabolites were increased by MISO. $AUC_{0-\infty}$

**Table II** The effect of 0.5 mg g$^{-1}$ MISO on the terminal half-life and plasma area under the curve ($AUC_{0-\infty}$) of CCNU metabolites following 20 mg kg$^{-1}$ CCNU. MISO was given 30 min before CCNU. Calculated from pooled data from 4 experiments.

| *Parameters* | *Pretreatment* | *Trans*-4 | *Cis*-4 | *Cis*-3 | *Trans*-3 | *Total* |
|---|---|---|---|---|---|---|
| Terminal $t_{\frac{1}{2}\beta^*}$ (min) | Control | 86.4 (67.6–113.8) | 91.7 (65.9–150.8) | 108.1 (95.0–125.4) | 64.3 (49.3–92.7) | 94.2 (72.4–134.8) |
| | MISO | 84.8 (71.0–105.2) | 76.7 (58.5–111.4) | 109.6 (88.7–143.5) | 68.2 (52.4–97.8) | 85.2 (67.9–109.0) |
| $AUC_{0-\infty}$ ($\mu g ml^{-1}$ min) | Control | 370 | 67 | 24 | 13 | 473 |
| | MISO | 778 | 109 | 32 | 16 | 936 |

*95% confidence limits in parentheses.

D

**Figure 4** The effect of $0.5\,mg\,g^{-1}$ MISO given 30 min before on the pharmacokinetics of the total nitrosoureas (**e**) and the 4 important monohydroxylated metabolites of CCNU after an i.p. dose of $20\,mg\,kg^{-1}$ of the parent compound: (**a**) *Trans* 4; (**b**) *Cis* 3; (**c**) *Cis* 4; (**d**) *Trans* 3. Closed symbols (◆, ●, ▲, ■) denote mice receiving CCNU alone, open symbols (◇, ○, △, □) denote mice with MISO pretreatment. The data were obtained in 4 independent experiments, indicated by different symbols. Each datum point is for 3 mice.

values were elevated by MISO, particularly for the *trans*-4 and *cis*-4 metabolites and the total nitrosoureas (Table II). However the terminal $t_{\frac{1}{2}\beta}$ values were not significantly altered by MISO ($P>0.1$) indicating that the main effects occurred during the production and initial clearance of the metabolites. It should be noted that the peak concentration of the total nitrosoureas was not affected by MISO (Figure 4) whereas the $AUC_{0-\infty}$ was increased by a factor of 2.

The lack of effects on the $\beta$-phase clearance is consistent with the hypothesis that the rate-limiting process of the $\beta$-phase plasma clearance of CCNU and metabolites is not hepatic metabolism but the redistribution from tissue depots as adipose tissue back into the plasma. A possible alternative explanation was that the concentration of MISO in plasma may have dropped below the inhibitory level during the $\beta$-phase. This was ruled out because in an experiment where $0.5\,mg\,g^{-1}$ MISO was given during the early $\beta$-phase of CCNU clearance (1 h after CCNU), there was again no significant change in the $t_{\frac{1}{2}\beta}$ ($P>0.1$, data not shown). However the apparent volume of distribution again appeared to be reduced.

### *CCNU dose modification by MISO*

Figure 5 shows the concentrations of total nitrosoureas in the plasma of mice given CCNU ($20\,mg\,kg^{-1}$) alone, CCNU ($20\,mg\,kg^{-1}$) with MISO

**Figure 5** The dose-modifying effect of $0.5\,mg\,g^{-1}$ of MISO given 30 min before on the plasma pharmacokinetics of nitrosoureas. ● $20\,mg\,kg^{-1}$ CCNU alone, ○ $20\,mg\,kg^{-1}$ with MISO pretreatment, ■ $40\,mg\,kg^{-1}$ CCNU alone. Each datum point is for 3 mice.

($0.5\,mg\,g^{-1}$, 0.5 h before) or CCNU ($40\,mg\,kg^{-1}$) alone. It can be seen that for the initial 60 min or so after CCNU the plasma level of cytotoxic nitrosoureas was substantially higher in the mice that received $40\,mg\,kg^{-1}$ CCNU than those that received $20\,mg\,kg^{-1}$ CCNU with MISO ($0.5\,mg\,kg^{-1}$) pretreatment. However, after this initial period very similar values were found. The two treatment regimes nevertheless gave very similar values for plasma total nitrosoureas $AUC_{0-\infty}$: $1090\,\mu g\,ml^{-1}$ min for the higher CCNU dose and $1000\,\mu g\,ml^{-1}$ min for the lower CCNU dose with MISO pretreatment, compared to $502\,\mu g\,ml^{-1}$ min for the lower dose alone. Thus the dose-modifying factor (DMF) for drug exposure by MISO was $\sim 2$, whereas the DMF for peak concentration was very much less.

### *The dose-response of MISO on CCNU clearance*

The plasma clearance of CCNU was estimated after varying MISO doses ($0.05\,mg\,g^{-1} - 1\,mg\,g^{-1}$) by monitoring its plasma concentrations at a fixed time (25 min after injection). Some typical results are shown in Figure 6A. Plasma clearance of CCNU was reduced as the MISO dose was increased, with a threshold MISO dose at about $0.3\,mg\,g^{-1}$.

The effects of varying the interval between $0.5\,mg\,g^{-1}$ MISO and CCNU administration are shown in Figure 6B. MISO was found to be most effective when given 0.5–1 h prior to CCNU. Its effectiveness was reduced with increasing interval up to 4 h, and no effects were observed at 6 h.

### *Effects of MISO on the CCNU concentrations in tumours*

Several experiments were carried out to determine the effect of MISO on the concentrations of CCNU in 3 transplantable mouse tumours (KHT, RIF-1 and EMT6) and the HT29 colon carcinoma xenograft. Figure 7 shows all the data obtained. The ratio of tumour/plasma CCNU concentrations was tumour dependent: KHT and HT29 gave relatively high ratios, EMT6 an intermediate, and RIF-1 a low ratio (Table III). Tumour/plasma ratios were not altered by $0.5\,mg\,g^{-1}$ MISO (Table III), but in all 4 tumours the CCNU concentrations were consistently higher after MISO. Similar differences were seen with the hydroxylated metabolites (data not shown).

More detailed experiments were carried out to determine the initial tumour uptake in the KHT tumours (Figure 7). It was found that the peak tumour concentration lagged considerably behind that of the peak plasma concentration suggesting a compromised uptake by tumour, due possibly to

**Figure 6(a)** The dose-response of MISO on the plasma concentration of CCNU 25 min after $20\,mg\,kg^{-1}$ CCNU. MISO was given 30 min before CCNU. (**b**) The effect of varying the interval between MISO ($0.5\,mg\,g^{-1}$) and CCNU ($20\,mg\,kg^{-1}$) administration on the plasma CCNU concentration 25 min after $20\,mg\,kg^{-1}$ CCNU. Each datum point is for 2 mice. Dotted lines represent the concentrations of CCNU in control mice.

**Table III** Tumour/plasma ratios of CCNU and total nitrosoureas 30 min after an i.p. dose of $20\,mg\,kg^{-1}$ CCNU with or without MISO pretreatment ($0.5\,mg\,g^{-1}$ 30 min before CCNU). Pooled data from several experiments.

| | | *Tumour/plasma ratio* | |
|---|---|---|---|
| *Tumour* | *Pretreatment* | *CCNU* | *Total nitrosoureas* |
| KHT | Control | 1.20±0.19 | 0.8 ±0.21 |
| | MISO | 1.30±0.35 | 0.86±0.18 |
| RIF-1 | Control | 0.30±0.08 | 0.24±0.05 |
| | MISO | 0.40±0.08 | 0.22±0.03 |
| EMT6 | Control | 0.58±0.12 | 0.55±0.14 |
| | MISO | 0.62±0.21 | 0.46±0.17 |
| HT29 | Control | 0.77±0.14 | 0.61±0.13 |
| | MISO | 1.03±0.19 | 0.70±0.20 |

Results shown are mean ±2 s.e. of 3–6 determinations.

inadequate blood supply. MISO increased the peak tumour concentration by a factor of ~2.4.

*Binding of CCNU to plasma protein*

Table IV shows the extent to which CCNU and its major metabolite, *trans*-4 monohydroxylated CCNU, bind to mouse plasma proteins. At concentrations up to $0.5\,mg\,ml^{-1}$ (2.5 mM) MISO did not appear to affect the binding of the nitrosoureas; this was found to be the case for both *in vivo* and *in vitro* binding.

**Table IV** *In vivo* and *in vitro* binding of CCNU and *trans*-4-hydroxy-CCNU to mouse plasma protein with and without the presence of $0.5\,mg\,ml^{-1}$ MISO. Calculated from pooled data from 2 experiments.

| | *Pretreatment* | *CCNU* | *trans-4-hydroxy-CCNU* |
|---|---|---|---|
| % bound *in vitro* ±2 s.e. | MISO | 94±2.5 | 53±2.2 |
| (n=8) | Control | 96±1.5 | 49±2.9 |
| % bound *in vivo* ±2 s.e. | MISO | 90±3.7 | 53±4.7 |
| (n=7) | Control | 93±2.1 | 51±3.4 |

None of the differences between MISO and control were statistically significant ($P>0.1$).

*Spontaneous chemical degradation of CCNU*

CCNU undergoes spontaneous degradation in aqueous solution to form non-u.v. absorbing species. We have compared the chemical half-lives of CCNU in 0.1 M PBS, pH 7.4, with and without $0.5\,mg\,ml^{-1}$ (2.5 mM) MISO. No significant difference was found ($P>0.1$); the $t_{\frac{1}{2}}$ values were 43 (39–47) min and 40 (36–46) min respectively.

### Discussion

The results of these studies clearly show that single high doses of MISO reduced the rate of plasma clearance of CCNU and its active metabolites. The tumour concentrations of CCNU generally reflect the plasma concentrations, and therefore MISO has the effect of increasing the tumour exposure to the cytotoxic nitrosoureas.*

The pharmacokinetics of CCNU, not described in such detail previously, appear to involve a rapid initial clearance due to metabolism to the

*It may be significant that the magnitude of the tumour/plasma ratios of the 3 murine tumours KHT, EMT6 and RIF-1 also reflect their *in vivo* sensitivity to CCNU.

**Figure 7** The effect of 0.5 mg g$^{-1}$ MISO on the concentration of CCNU in (**a**) KHT, (**b**) RIF-1, (c) EMT6 and (**d**) HT29 tumours. Closed symbols 20 mg kg$^{-1}$ CCNU alone. Open symbols 20 mg kg$^{-1}$ CCNU with 0.5 mg g$^{-1}$ MISO 30 min before. Different symbols represent independent experiments. Each datum point is for 2 or 3 tumours.

hydroxylated metabolites; this is followed by a slower terminal clearance probably limited by slow release of the highly lipophilic CCNU from hydrophobic depots, such as adipose tissue. The initial kinetics of the increased concentrations of CCNU and its hydroxylated metabolites are consistent with a model in which MISO inhibits the hydroxylation of CCNU and may also inhibit the subsequent metabolism of the hydroxylated derivatives to unknown species. The increased levels of CCNU at later times were due to a reduction in the volume of distribution, and a similar effect may also occur with the metabolites. The mechanism by which MISO decreases the apparent volume of distribution of CCNU is not known, but may involve a reduced penetration of the drug into, or a reduced retention by, certain body tissue depots. Both MISO (Shoemaker *et al.*, 1982) and the nitrosoureas (Walker & Hilton 1976) are metabolized by the liver mixed function oxidase enzymes and it is likely that MISO may act as a competitive inhibitor of nitrosourea metabolism. Experiments are in progress to determine the nature of the inhibition in liver microsomal preparations. The inhibition is not due to the hypothermia seen with higher doses of MISO (Gomer & Johnson, 1979), since the dose used in the present study does not alter the rectal temperature significantly. Effects of MISO on protein binding and spontaneous chemical degradation were also excluded by experiments reported here.

Several authors have demonstrated selective enhancement of murine tumour toxicity of CCNU by large single doses of MISO and a therapeutic gain is consistently observed (Hirst *et al.*, 1982;

Siemann, 1981, 1982*b*; Twentyman & Workman 1982). The mechanism of action of MISO chemosensitisation is not fully understood, but in view of the increase in the plasma and tumour levels of cytotoxic nitrosoureas by $0.5\,mg\,g^{-1}$ MISO it is likely that pharmacokinetic modification is an important contributory mechanism. In support of this we found that the MISO dose needed to produce significant reduction of CCNU clearance had to be $\geqslant 0.3\,mg\,g^{-1}$, and this correlates well with the doses required for effective chemosensitisation. The effect of the timing of the two drugs is also similar for pharmacokinetic changes and chemosensitization.

Several other lines of evidence support the pharmacokinetic mechanism. SKF-525-A, the classical inhibitor of xenobiotic detoxification enzymes, has been shown to produce excellent enhancement of CCNU cytotoxicity (Workman & Twentyman 1982; Siemann, 1983). We have also demonstrated a therapeutic gain for this combination (Workman & Twentyman, in preparation), but Siemann (1983) found that the normal tissue toxicity was also increased. Other studies in our laboratory have clearly demonstrated inhibition of drug metabolising enzymes by MISO (Workman *et al.*, 1983), and we have also shown that MISO slows the clearance of chlorambucil and melphalan (Workman *et al.*, 1983, Lee & Workman unpublished). Other laboratories have found that MISO reduces the clearance of melphalan (Clutterbuck *et al.*, 1982; McNally *et al.*, personal communication) and the active metabolites of cyclophosphamide and BCNU (Tannock, 1980; McNally *et al.*, personal communication).

Our results on the pharmacokinetics of CCNU provide a possible explanation for the therapeutic gain with the combination of MISO and CCNU. MISO increased the peak tumour concentration of CCNU and total nitrosoureas without increasing the peak plasma concentration. This was due to the fact that peak tumour levels lag behind the peak plasma levels, probably because of inadequate tumour blood supply. If the peak nitrosourea concentration is more important for cytotoxicity than overall exposure, and if the better perfused dose-limiting normal tissues (gut and bone marrow) follow the plasma concentration more closely than the tumour, then the differential effect of MISO will result in a therapeutic gain. We are now developing methods to assay nitrosoureas in bone marrow and gut.

Differences in the pharmacokinetics of various nitrosoureas may provide an alternative explanation for the interesting chemosensitization data of Mulcahy (1982) who reported a good correlation between the dose-modifying factor produced by pretreatment with MISO and the relative carbamoylating activities of individual nitrosoureas. However, the order of relative carbamoylating activities of the nitrosoureas studied also correlates with increasing relative partition coefficient which generally determines whether or not a drug is metabolized by hepatic enzymes.

The important question is whether the MISO effect can be obtained with clinically relevant doses. The doses of MISO used in experimental studies have generally been in the range of $0.3–1\,mg\,g^{-1}$ ($1.5–5\,mmol\,kg^{-1}$) giving peak plasma concentrations of $300–1000\,\mu g\,ml^{-1}$ (1.5–5 mM). In the clinic the largest single dose normally given to patients ($3\,g\,m^{-2}$) produces a peak plasma concentration of only $100\,\mu g\,ml^{-1}$ (0.5 mM) (Workman, 1980*b*). However, the plasma half-life of MISO in man is 10–20 times longer than in the mouse (Workman, 1980*b*). We have therefore tried to mimic human pharmacokinetics in mice by giving multiple small doses of MISO (Brown & Hirst, 1982). Preliminary results have indicated no appreciable change in CCNU pharmacokinetics when plasma MISO concentrations were maintained at about $90\,\mu g\,ml^{-1}$ for over 4 h prior to CCNU treatment (Lee & Workman unpublished) and this probably explains our observed lack of chemosensitization with this regime (Twentyman & Workman, 1983). In contrast, Brown & Hirst (1982) did observe chemosensitization with this regime, and the disparity may be due to the proximity of the MISO plasma concentration to the threshold for changes in pharmacokinetics and chemosensitization. In experiments in the dog, which is a better model for human MISO metabolism (White *et al.*, 1979), a relatively low dose of MISO ($150\,mg\,kg^{-1}$ i.v.) given immediately before CCNU ($5\,mg\,kg^{-1}$ i.v.) has produced significant changes in the clearance of CCNU and its metabolites (Lee *et al.*, unpublished).

We have recently found that the HPLC method can be used to determine the pharmacokinetics of CCNU and its metabolites in man, and this is now being used to investigate the pharmacokinetic interactions between nitroimidazoles and CCNU in patients.

It should be emphasized that while the alterations in CCNU pharmacokinetics by MISO described here appear to explain the therapeutic gain for this combination, we have at present no comparable detailed data to support a similar mechanism for the therapeutic gain seen with other cytotoxic agents. We feel that poor penetration of cytotoxic drugs into tumours at early times may be involved, and experiments are in progress to test this.

We wish to thank Prof. N.M. Bleehen for his support and Dr. P.R. Twentyman for valuable discussions. We acknowledge the generosity of Dr. C.E. Smithen of Roche

Products Ltd. (Welwyn) for the supplies of MISO; Dr. T.P. Johnston of the Southern Research Institute (Alabama, U.S.A.) for the synthetic CCNU metabolites; and Lundbeck and Dr. Ven Narayanan of the N.C.I. for CCNU. We also thank S. Saxby for providing the HT29 xenografts, Ms. J. Donaldson for excellent technical assistance and Mrs. B. Smith for typing the manuscript.

### References

BROWN, J.M. (1982). The mechanisms of cytotoxicity and chemosensitisation by misonidazole and other nitroimidazoles. *Int. J. Radiat. Oncol. Biol. Phys.*, **8**, 675.

BROWN, J.M. & HIRST, D.G. (1982). The effect of clinically achievable levels of misonidazole on the response of tumour and normal tissues in the mouse to alkylating agents. *Br. J. Cancer*, **45**, 700.

CLUTTERBUCK, R.D., MILLAR, J.L. & MCELWAIN, T.J. (1982). Misonidazole enhancement of the action of BCNU and melphalan against human melanoma xenografts. *Am. J. Clin. Oncol.*, **5**, 73.

GOMER, C.J. & JOHNSON, R.J. (1979). Relationship between misonidazole toxicity and core temperature in C3H mice. *Radiat. Res.*, **78**, 329.

HILTON, J. & WALKER, M.D. (1975). Hydroxylation of 1-(2-chloroethyl)-3-cyclohexyl-1-nitrosourea. *Biochem. Pharmacol.*, **24**, 2153.

HIRST, D.G., BROWN, J.M. & HAZELHURST, J.L. (1982). Enhancement of CCNU cytoxicity by misonidazole. Studies of the therapeutic ratio and possible mechanisms. *Br. J. Cancer*, **46**, 109.

MCNALLY, N.J. (1982). Enhancement of chemotherapy agents. *Int. J. Radiat. Oncol. Biol. Phys.*, **8**, 593.

MILLAR, B.C. (1982). Hypoxic cell radiosensitizers as potential adjuvants to conventional chemotherapy for the treatment of cancer. *Biochem. Pharmacol.*, **31**, 2439.

MONTGOMERY, J.A., JOHNSTON, P.J., THOMAS, H.J., PIPER, J.R. & TEMPLE, C.Jr. (1977). *Adv. Chromatogr.*, **15**, p. 169.

MULCAHY, R.T. (1982). Chemical properties of nitrosoureas: implications for interaction with misonidazole. *Int. J. Radiat. Oncol Biol. Phys.*, **8**, 599.

MULCAHY, R.T., SIEMANN, D.W. & SUTHERLAND, R.M. (1981). *In vivo* response of KHT sarcomas to combination chemotherapy with radiosensitizers and BCNU. *Br. J. Cancer*, **43**, 93.

SHOEMAKER, D.D., MCMANUS, M.E., HOERANF, R. & STRONG, J.M. (1982). Studies on the O-demethylation of misonidazole by rat liver microsomes. *Cancer Treat. Rep.*, **66**, 1343.

SIEMANN, D.W. (1981). *In vivo* combination of misonidazole and the chemotherapeutic agent CCNU. *Br. J. Cancer*, **43**, 367.

SIEMANN, D.W. (1982*a*). Potentiation of chemotherapy by hypoxic cell radiation sensitizers—A review. *Int. J. Radiat. Oncol. Biol. Phys.*, **8**, 1029.

SIEMANN, D.W. (1982*b*). Response of murine tumours to combinations of CCNU with misonidazole and other radiation sensitizers. *Br. J. Cancer*, **45**, 272.

SIEMANN, D.W. (1983). The effect of pretreatment with phenobarbitone or SKF 525A on the toxicity and antitumour activity of CCNU. *Cancer Treat. Rep.* (in press).

TANNOCK, I.F. (1980). *in vivo* interaction of anti-cancer drugs with misonidazole or metronidazole: Cyclophosphamide and BCNU. *Br. J. Cancer*, **42**, 871.

TWENTYMAN, P. & WORKMAN, P. (1982). Effect of misonidazole or metronidazole pretreatment on the response of the RIF-1 mouse sarcoma to melphalan, cyclophosphamide, chlorambucil and CCNU. *Br. J. Cancer*, **45**, 447.

TWENTYMAN, P.R. & WORKMAN, P. (1983). An investigation of the possibility of chemosensitization by clinically achievable concentrations of misonidazole. *Br. J. Cancer*, **47**, 187.

TWENTYMAN, P.R., KALLMAN, R.F. & BROWN, J.M. (1979). The effect of time between X-irradiation and chemotherapy on the growth of three solid mouse tumours—I. Adriamycin. *Int. J. Radiat. Oncol. Biol. Phys.*, **5**, 1255.

WALKER, M.D. & HILTON, J.H. (1976). Nitrosourea pharmacodynamics in relation to the central nervous system. *Cancer Treat. Rep.*, **60**, 725.

WARENIUS, H.M. & BLEEHEN, N.M. (1982). *In vivo–in vitro* clonogenic assays in a human tumour xenograft with a high plating efficiency. *Br. J. Cancer*, **46**, 45.

WHEELER, G.P., JOHNSTON, T.P., BOWDON, B.J., MCCALEB, G.S., HILL, D.L. & MONTGOMERY, J.A. (1977). Comparison of the properties of metabolites of CCNU. *Biochem. Pharmacol.*, **26**, 2331.

WHITE, R.A.S. & WORKMAN, P. (1980). Pharmacokinetic and tumour penetration properties of the hypoxic cell radiosensitizer desmethylmisonidazole (Ro 05-9963) in dogs. *Br. J. Cancer*, **41**, 268.

WHITE, R.A.S., WORKMAN, P., FREEDMAN, L.S., OWEN, L.N. & BLEEHEN, N.M. (1979). The pharmacokinetics of misonidazole in the dog. *Eur. J. Cancer*, **15**, 1233.

WORKMAN, P. (1980*a*). Dose-dependence and related studies on the pharmacokinetics of misonidazole and desmethylmisonidazole in mice. *Cancer Chemother. Pharmacol.*, **5**, 27.

WORKMAN, P. (1980*b*). Pharmacokinetics of hypoxic cell radiosensitisers—A review. *Cancer Clin. Trials*, **3**, 237.

WORKMAN, P. & BROWN, J.M. (1981). Structure-pharmacokinetic relationships for misonidazole analogues in mice. *Cancer Chemother. Pharmacol.*, **6**, 39.

WORKMAN, P. & TWENTYMAN, P.R. (1982). Enhancement by electron-affinic agents of the therapeutic effects of cytotoxic agents against the KHT tumour: structure-activity relationships. *Int. J. Radiat. Oncol. Biol. Phys.*, **8**, 623.

WORKMAN, P., LITTLE, C.J., MARTEN, T.R. & 4 others (1978). Estimation of the hypoxic cell-sensitiser misonidazole and its O-demethylated metabolite in biological materials by reversed-phase high-performance liquid chromatography. *J. Chromatogr.*, **145**, 507.

WORKMAN, P., TWENTYMAN, P.R., LEE, F.Y.F. & WALTON, M. (1983). Drug metabolism and chemosensitization: nitroimidazoles as inhibitors of drug metabolism. *Biochem. Pharmacol.*, **32**, 857.


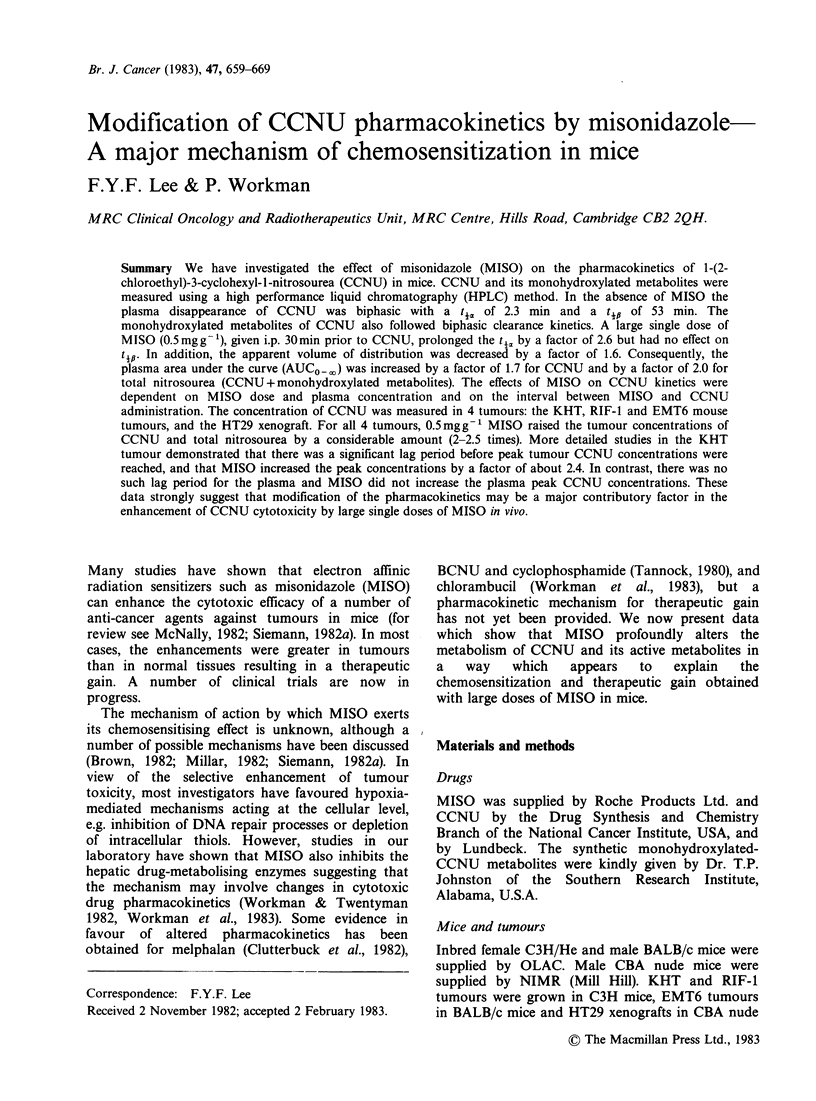

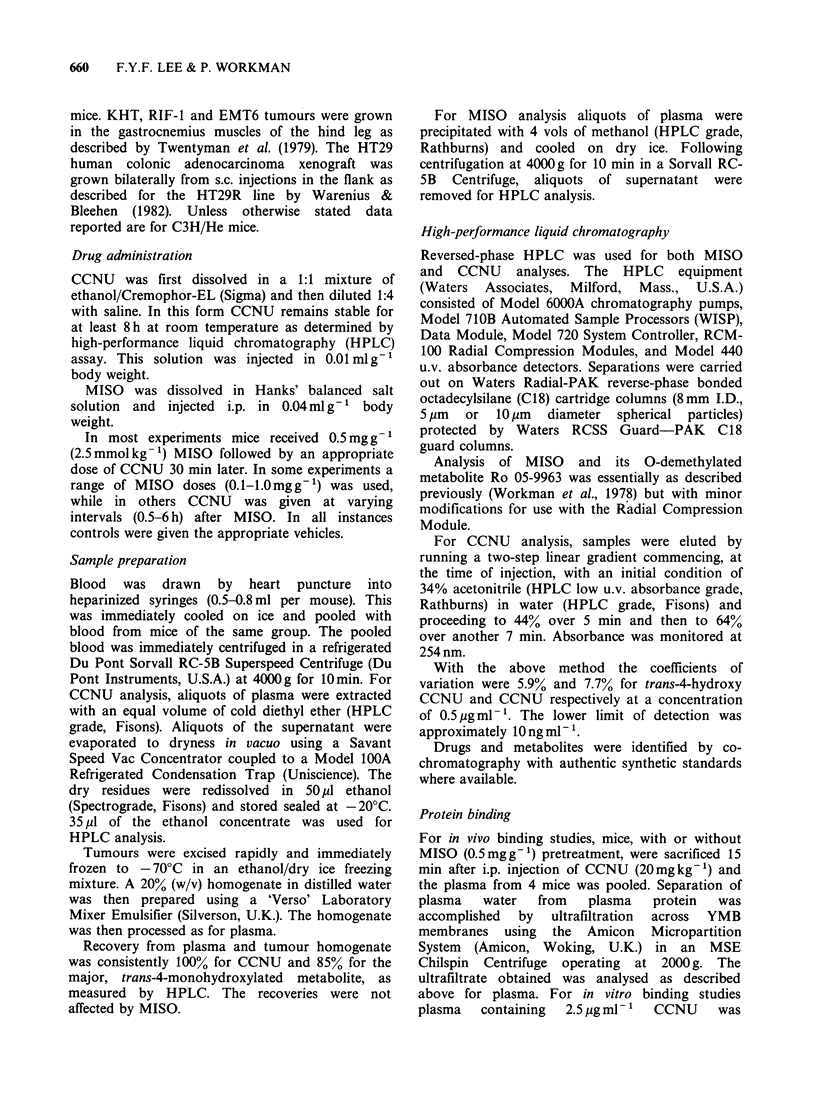

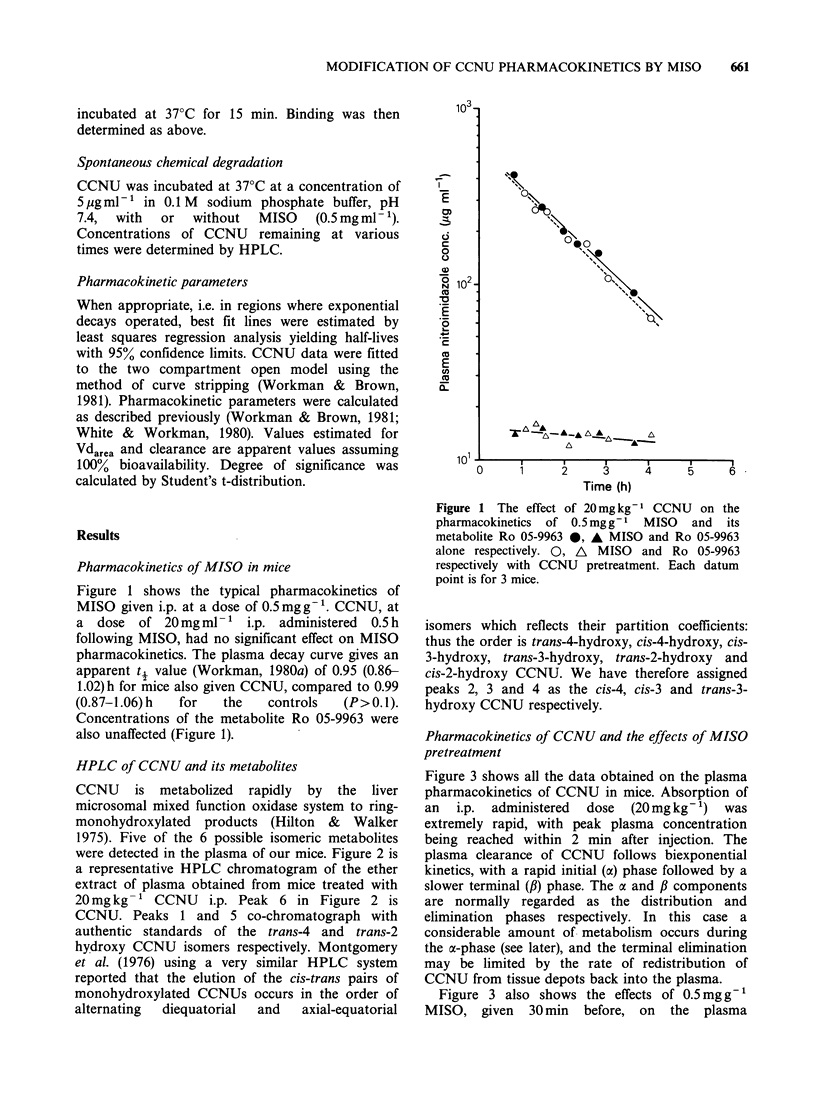

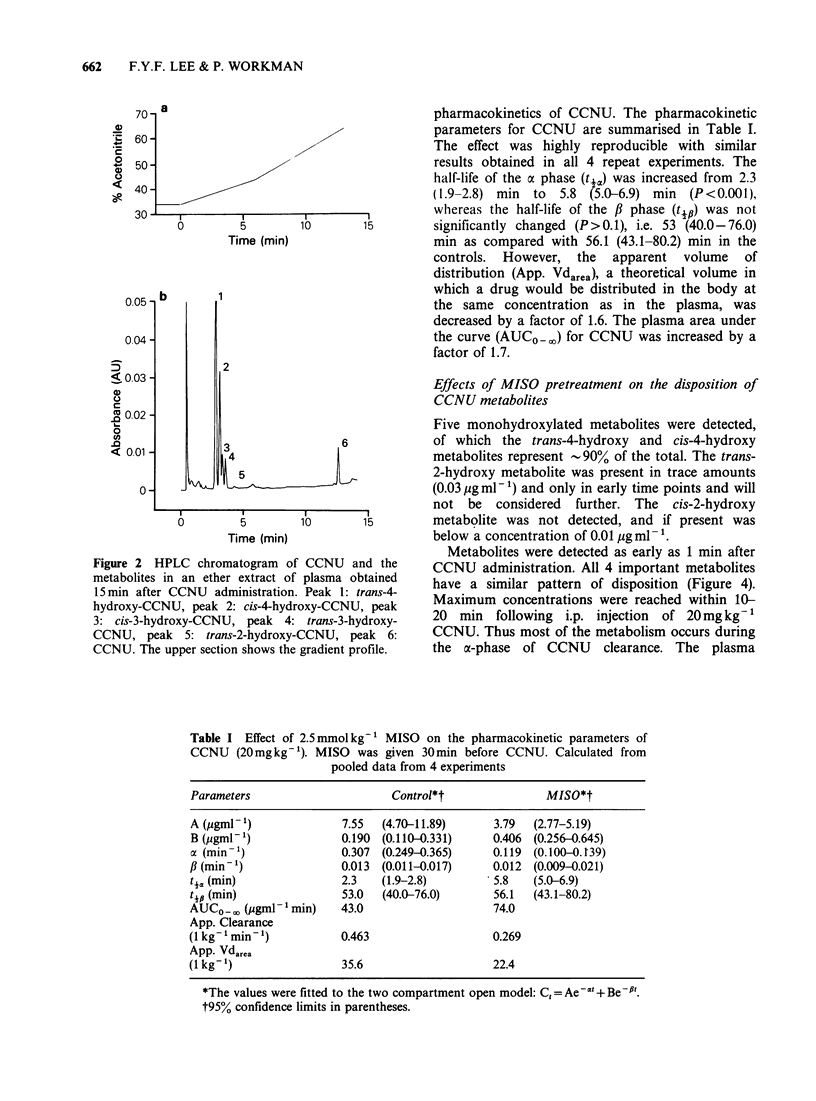

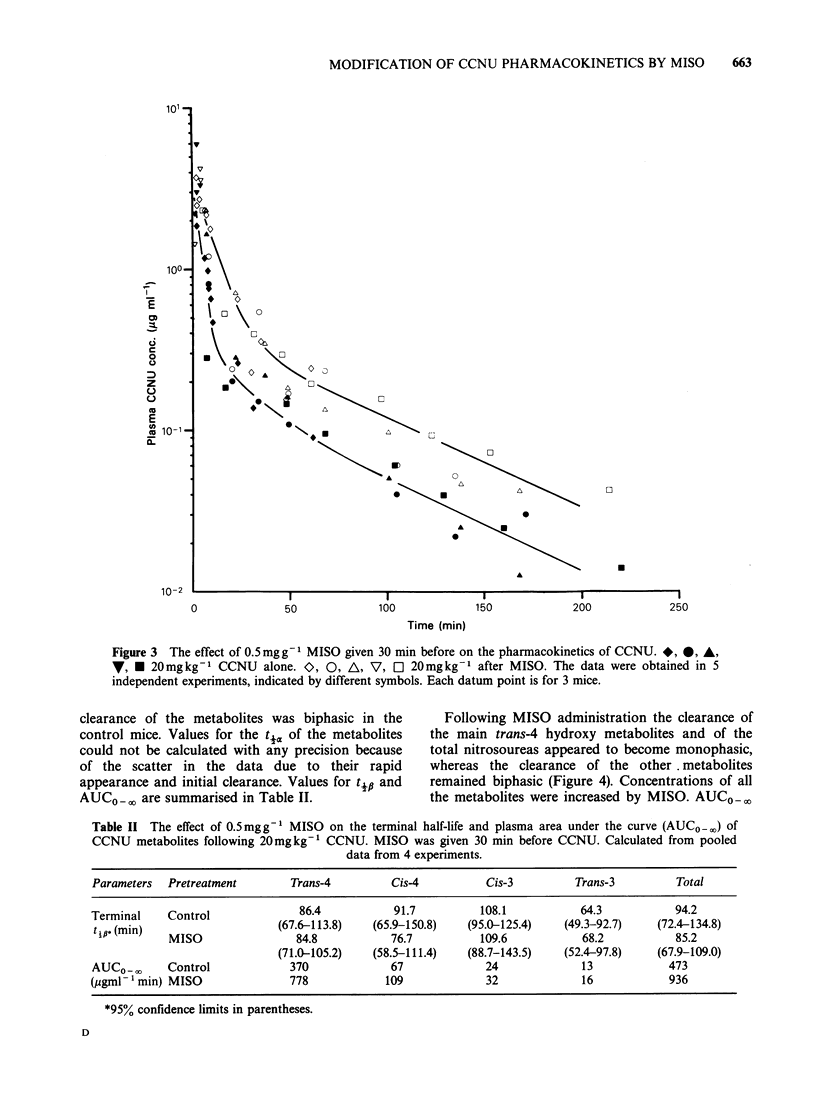

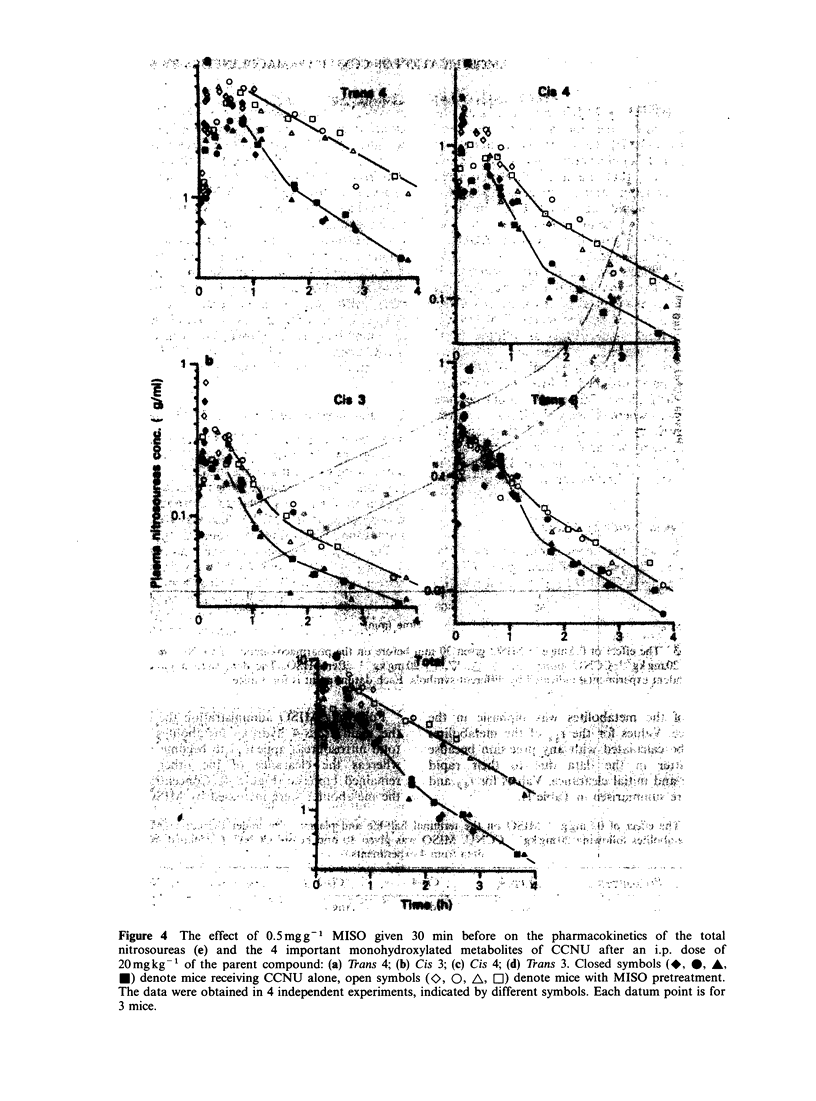

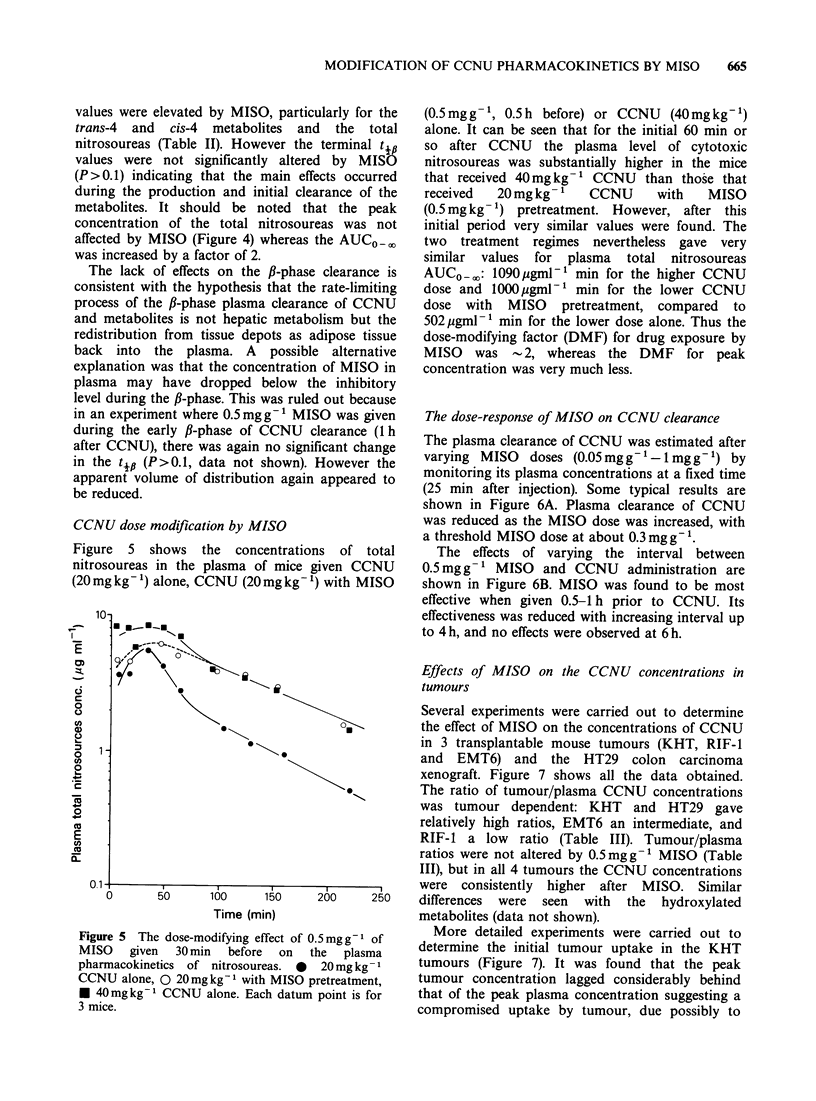

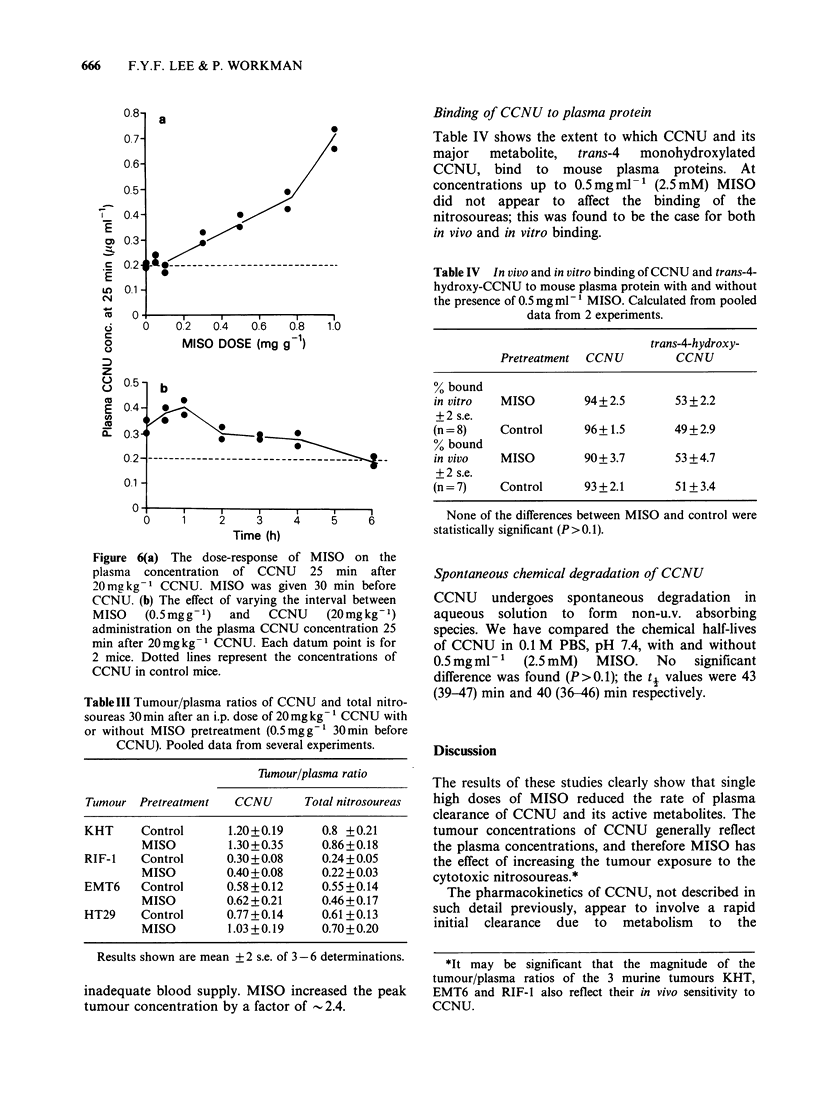

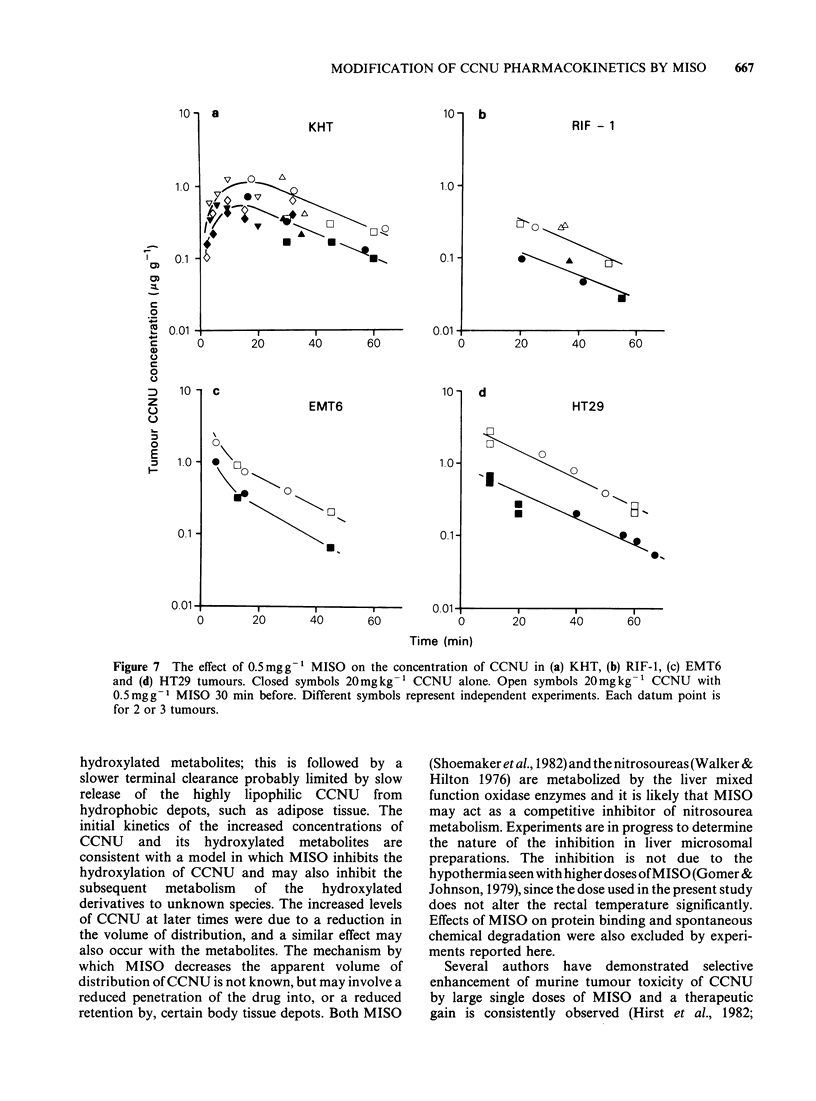

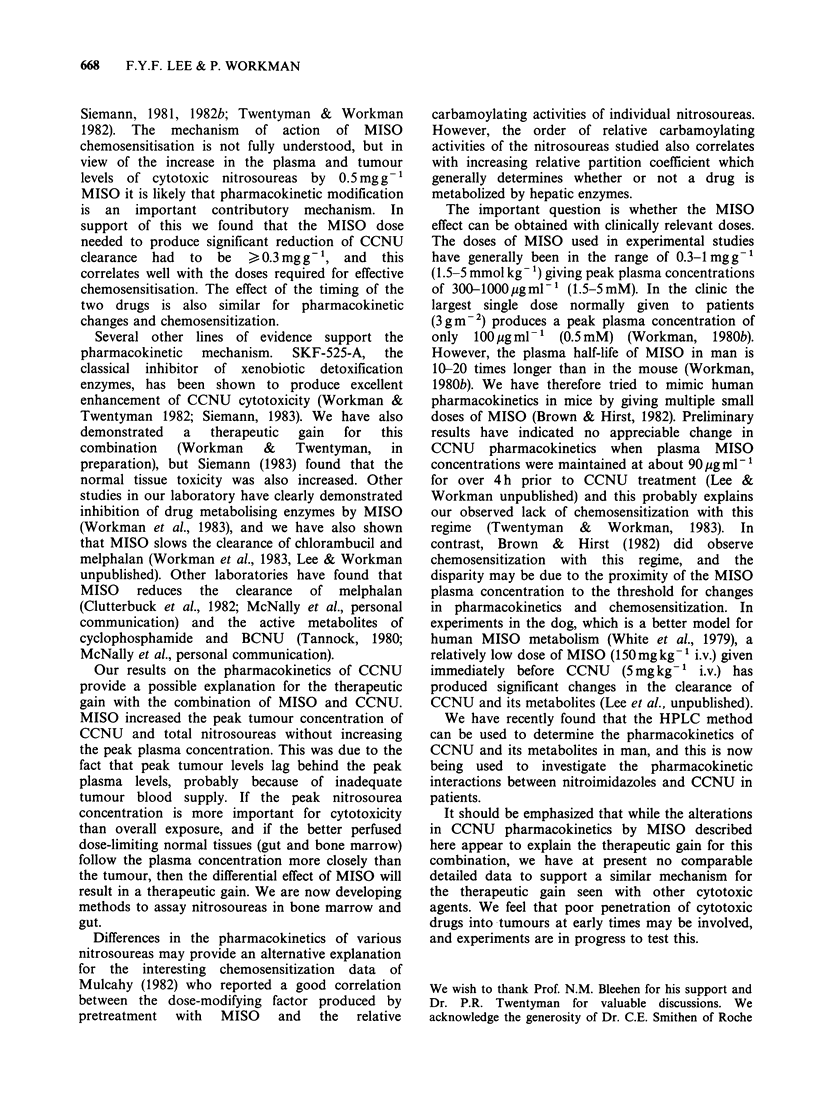

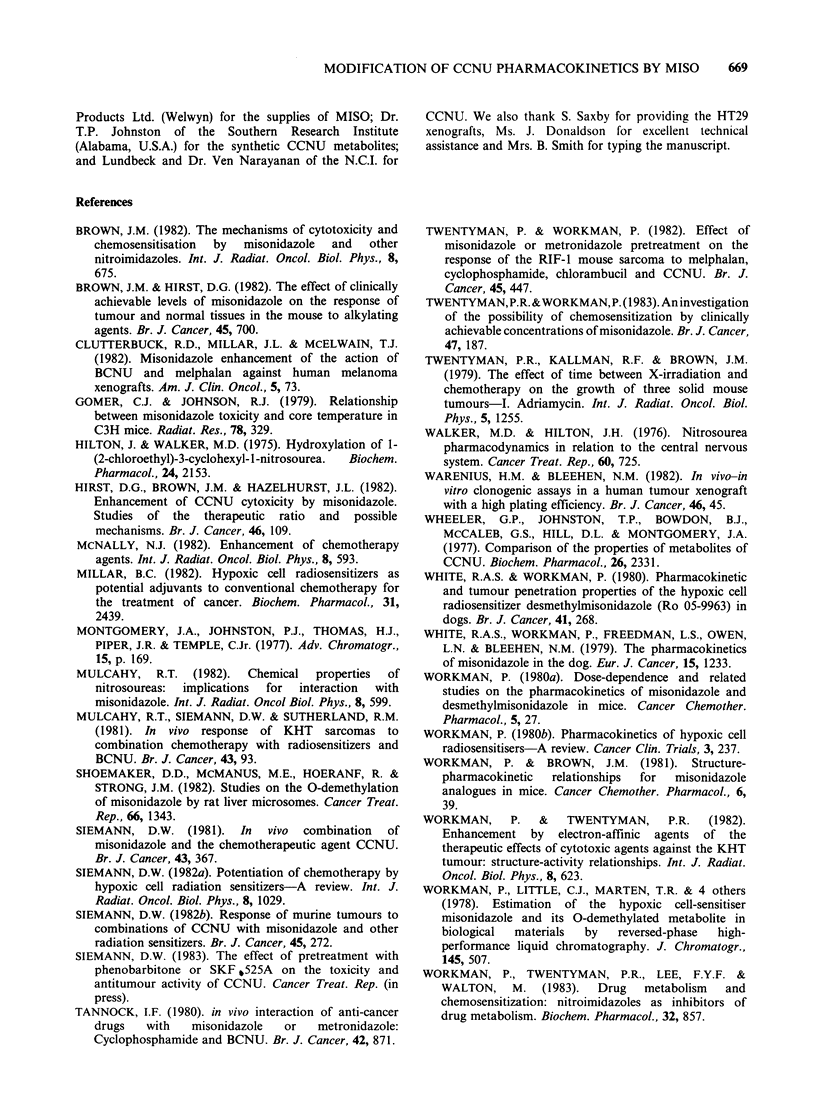


## References

[OCR_01305] Brown J. M., Hirst D. G. (1982). Effect of clinical levels of misonidazole on the response of tumour and normal tissues in the mouse to alkylating agents.. Br J Cancer.

[OCR_01299] Brown J. M. (1982). The mechanisms of cytotoxicity and chemosensitization by misonidazole and other nitroimidazoles.. Int J Radiat Oncol Biol Phys.

[OCR_01311] Clutterbuck R. D., Millar J. L., McElwain T. J. (1982). Misonidazole enhancement of the action of BCNU and melphalan against human melanoma xenografts.. Am J Clin Oncol.

[OCR_01317] Gomer C. J., Johnson R. J. (1979). Relationship between misonidazole toxicity and core temperature in C3H mice.. Radiat Res.

[OCR_01322] Hilton J., Walker M. D. (1975). Hydroxylation of 1-(2-chloroethyl)-3-cyclohexyl-1-nitrosourea.. Biochem Pharmacol.

[OCR_01327] Hirst D. G., Brown J. M., Hazlehurst J. L. (1982). Enhancement of CCNU cytotoxicity by misonidazole: possible therapeutic gain.. Br J Cancer.

[OCR_01333] McNally N. J. (1982). Enhancement of chemotherapy agents.. Int J Radiat Oncol Biol Phys.

[OCR_01337] Millar B. C. (1982). Hypoxic cell radiosensitizers as potential adjuvants to conventional chemotherapy for the treatment of cancer.. Biochem Pharmacol.

[OCR_01343] Montgomery J. A., Johnston T. P., Thomas H. J., Piper J. R., Temple C. (1977). The use of microparticulate reversed-phase packing in high-pressure liquid chromatography of compounds of biological interest.. Adv Chromatogr.

[OCR_01348] Mulcahy R. T. (1982). Chemical properties of nitrosoureas: implications for interaction with misonidazole.. Int J Radiat Oncol Biol Phys.

[OCR_01353] Mulcahy R. T., Siemann D. W., Sutherland R. M. (1981). In vivo response of KHT sarcomas to combination chemotherapy with radiosensitizers and BCNU.. Br J Cancer.

[OCR_01359] Shoemaker D. D., McManus M. E., Hoerauf R., Strong J. M. (1982). Studies on the O-demethylation of misonidazole by rat liver microsomes.. Cancer Treat Rep.

[OCR_01365] Siemann D. W. (1981). In vivo combination of misonidazole and the chemotherapeutic agent CCNU.. Br J Cancer.

[OCR_01370] Siemann D. W. (1982). Potentiation of chemotherapy by hypoxic cell radiation sensitizers--a review.. Int J Radiat Oncol Biol Phys.

[OCR_01375] Siemann D. W. (1982). Response of murine tumours to combinations of CCNU with misonidazole and other radiation sensitizers.. Br J Cancer.

[OCR_01386] Tannock I. F. (1980). In vivo interaction of anti-cancer drugs with misonidazole or metronidazole: cyclophosphamide and BCNU.. Br J Cancer.

[OCR_01404] Twentyman P. R., Kallman R. F., Brown J. M. (1979). The effect of time between X-irradiation and chemotherapy on the growth of three solid mouse tumors--I. Adriamycin.. Int J Radiat Oncol Biol Phys.

[OCR_01398] Twentyman P. R., Workman P. (1983). An investigation of the possibility of chemosensitization by clinically achievable concentrations of misonidazole.. Br J Cancer.

[OCR_01391] Twentyman P., Workman P. (1982). Effect of misonidazole or metronidazole pretreatment on the response of the RIF-1 mouse sarcoma to melphalan, cyclophosphamide, chlorambucil and CCNU.. Br J Cancer.

[OCR_01411] Walker M. D., Hilton J. (1976). Nitrosourea pharmacodynamics in relation to the central nervous system.. Cancer Treat Rep.

[OCR_01416] Warenius H. M., Bleehen N. M. (1982). In vivo-in vitro clonogenic assays in a human tumour xenograft with a high plating efficiency.. Br J Cancer.

[OCR_01421] Wheeler G. P., Johnston T. P., Bowdon B. J., McCaleb G. S., Hill D. L., Montgomery J. A. (1977). Comparison of the properties of metabolites of CCNU.. Biochem Pharmacol.

[OCR_01433] White R. A., Workman P., Freedman L. S., Owen L. N., Bleehen N. M. (1979). The pharmacokinetics of misonidazole in the dog.. Eur J Cancer.

[OCR_01427] White R. A., Workman P. (1980). Pharmacokinetic and tumour-penetration properties of the hypoxic cell radiosensitizer desmethylmisonidazole (Ro 05-Ro-9963) in dogs.. Br J Cancer.

[OCR_01448] Workman P., Brown J. M. (1981). Structure-pharmacokinetic relationships for misonidazole analogues in mice.. Cancer Chemother Pharmacol.

[OCR_01438] Workman P. (1980). Dose-dependence and related studies on the pharmacokinetics of misonidazole and desmethylmisonidazole in mice.. Cancer Chemother Pharmacol.

[OCR_01461] Workman P., Little C. J., Marten T. R., Dale A. D., Ruane R. J., Flockhart I. R., Bleehen N. M. (1978). Estimation of the hypoxic cell-sensitiser misonidazole and its O-demethylated metabolite in biological materials by reversed-phase high-performance liquid chromatography.. J Chromatogr.

[OCR_01444] Workman P. (1980). Pharmacokinetics of hypoxic cell radiosensitizers: a review.. Cancer Clin Trials.

[OCR_01454] Workman P., Twentyman P. R. (1982). Enhancement by electron-affinic agents of the therapeutic effects of cytotoxic agents against the KHT tumor: structure-activity relationships.. Int J Radiat Oncol Biol Phys.

[OCR_01469] Workman P., Twentyman P. R., Lee F. Y., Walton M. I. (1983). Drug metabolism and chemosensitization. Nitroimidazoles as inhibitors of drug metabolism.. Biochem Pharmacol.

